# Low friction of metallic multilayers by formation of a shear-induced alloy

**DOI:** 10.1038/s41598-019-45734-7

**Published:** 2019-07-01

**Authors:** Ebru Cihan, Heike Störmer, Harald Leiste, Michael Stüber, Martin Dienwiebel

**Affiliations:** 1 Institute for Applied Materials - Computational Materials Science (IAM-CMS), Karlsruhe Institute of Technology (KIT), 76131 Karlsruhe, Germany; 2 Laboratory for Electron Microscopy (LEM), Karlsruhe Institute of Technology (KIT), 76131 Karlsruhe, Germany; 3 Institute for Applied Materials – Applied Materials Physics (IAM-AWP), Karlsruhe Institute of Technology (KIT), 76344 Eggenstein-Leopoldshafen, Germany; 4 Fraunhofer Institute for Mechanics of Materials (IWM), MicroTribology Center μTC, 79108 Freiburg, Germany

**Keywords:** Mechanical engineering, Metals and alloys

## Abstract

During sliding of metallic surfaces, the near surfaces undergo significant changes in terms of topography, composition and microstructure. Since friction and wear behavior of the materials are strongly influenced by sub-surface deformations, it is fundamental to investigate these effects. Therefore, the present study aims towards a better understanding of the behavior of friction depending on well-defined initial microstructures. By performing sliding experiments on Au-Ni multilayer samples under ultrahigh vacuum (UHV) conditions, we observe that the individual layer thickness of multilayer systems has a strong influence on friction behavior due to the transition in the dominant deformation mechanism near the surface. The experiments reported here provide a new route for lowering the friction force of metallic material systems in dry contact by providing more stable microstructures and alloy formation. Through ultrafine grains present in the alloy formed by mechanical mixing the number of grain boundaries strongly increases and hence, grain boundary-mediated deformation results in the low friction coefficient.

## Introduction

Most machinery is constructed from technical alloys and even a slight reduction of friction and wear of their sliding parts has a huge economic impact. However it is extremely difficult to predict friction of metallic surfaces because when they are subjected to sliding friction, they develop new microstructures, phases or composition that are not present in the original material at the near surface^1–3^. The development of these so-called ‘third bodies’ or tribomaterials^4^ strongly influences the frictional and wear behavior of tribological systems and many examples exist for technical alloys and pure metals^5–15^.

Previous studies suggested that mechanical mixing process during sliding is possible due to the formation of vortex-like structures^16–18^ in the tribomaterial. The process for producing vortex to drive mechanical mixing was described as a plastic flow process rather than a simple thermal diffusion^1^. Shear instabilities like in the case of the Kelvin-Helmholtz instability in fluid dynamics have previously been discussed to be responsible for the formation of vorticity when localized, inhomogeneous strains are generated among the surfaces in contact^19^. This description considers the material as homogeneous without having a microstructure and thus best represents amorphous materials (i.e. glasses). For crystalline materials, the mechanism of mechanical mixing depends on the microstructure^20^. It is likely that this process results in an increase in hardness due to the refinement of grains (Hall-Petch) whereas ductility diminishes^21^. However, there have been several studies^8,9,22–25^ where breakdown of the Hall-Petch effect was demonstrated when a transition in the dominant deformation mechanism is observed when a critical grain size is reached. The evolution of plastic deformation in face centered cubic (FCC) metals (i.e. Au and Ni) is mainly controlled by grain boundary- and dislocation-mediated plasticity depending on the size of grains as well as stress acting on the surface. It has been previously proposed that the transition in deformation mechanisms from dislocation- to grain boundary-mediated plasticity leads to a lower friction coefficient^8,24,25^. Sometimes, mechanical mixing processes driven by large plastic strains on sub-surfaces can be reinforced by mechanical alloying^26^.

In the study presented here, different Au-Ni metallic multilayer systems were prepared in order to systematically study the influence of their initial microstructures on friction behavior by means of sliding experiments with a ruby counter body under well-defined ultrahigh vacuum (UHV) conditions. The control of the parameters such as hardness, thickness and surface roughness of thin films^27^ have been carefully taken into account since they have profound impact on the formation of the third body during sliding of contact surfaces. Although the deformation mechanisms can be quite complex, multilayer thin films are extremely promising for studying these mechanisms and can serve as model materials for tribological purposes to be able to clarify distinctive deformation processes which have strong relation with their friction behavior as we aim to demonstrate in this article.

## Results

### Characterization of the as-grown Au-Ni multilayers

In order to identify the crystallographic features of the Au-Ni multilayers, X-ray diffraction (XRD) analysis has been performed. We confirmed the presence of Au (111) crystallographic planes and possible Ni (111) planes which have similar diffraction angle with the (200) order of Au (see Supplementary Fig. S1). Furthermore, we carried out atomic force microscope (AFM) experiments to characterize the topography of the as-grown multilayer samples in order to see the effect of layer thickness on surface roughness, and it has been observed that the roughness increases only slightly with increasing layer thickness (see Supplementary Fig. S2). Following this, the hardness of the multilayer samples has been measured via nanoindentation. We observed that an increase in the layer thickness leads to decrease in hardness of the multilayers by 58.3% ± 7%, from 10 to 100 nm sample, which is also in agreement with the Hall-Petch relationship (see Supplementary Fig. S3). Note however that a significant pile-up has been observed in our measurements; and also, the critical indentation depth should vary for each multilayer sample due to different layer thicknesses having distinct hardness.

**Figure 1:**
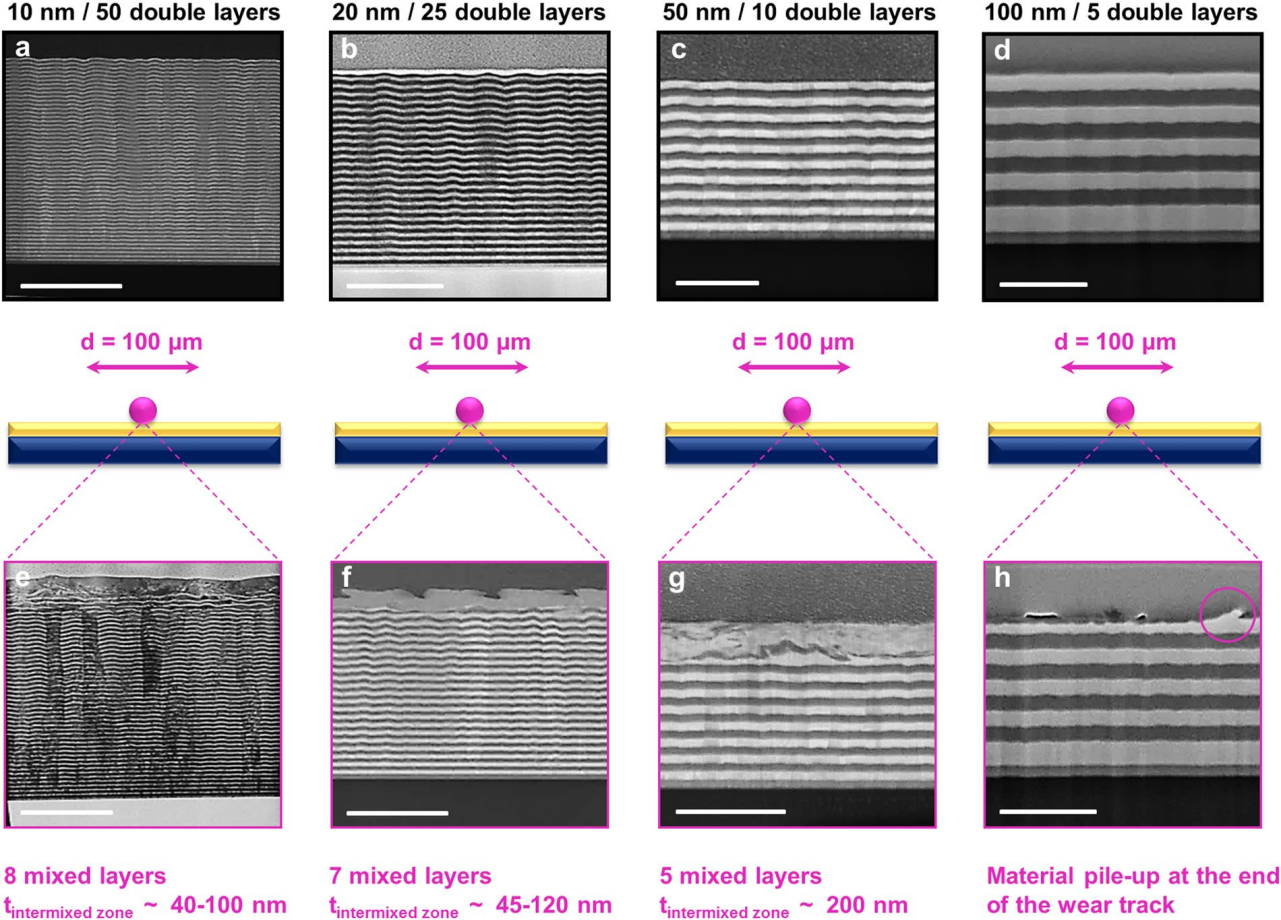
Characterization of the as-grown as well as worn Au-Ni multilayers. HAADF STEM cross-sections of the as-grown Au-Ni multilayers for an individual layer thickness of **(a)** 10 nm; (**b)** 20 nm; **(c)** 50 nm; **(d)** 100 nm. HAADF STEM cross-sections of the worn Au-Ni multilayers for an individual layer thickness of **(e)** 10 nm; **(f)** 20 nm; **(g)** 50 nm; **(h)** 100 nm after sliding for 100 cycles. The sliding process concludes with fully mixing (**e–f**), partially mixing (**g**), and material pile-up (only the uppermost Au layer is influenced) (**h**), respectively. Scale bars, 500 nm.

In order to prevent the structure from possible oxidation, the multilayer has been chosen such that the topmost layer is Au whereas the first layer on the substrate is Ni for all the studied samples. For the cross-sectional microstructure characterization of the as-grown multilayers, samples have been prepared via focused ion beam (FIB) milling following common rules for the lift-out technique^28^. The high annular angle dark field (HAADF) scanning transmission electron microscopy (STEM) cross-section images in Fig. 1a–d depict these original microstructures of Au-Ni multilayer systems with a layer thickness of 10 nm, 20 nm, 50 nm and 100 nm, respectively. Starting from a layer thickness of 10 nm, waviness of multilayers is seen in between Au and Ni layers due to residual strains possibly induced by the lattice mismatch between these metals which is about 15%, as well as the orientation dependent growth rate. At increasing layer thickness, this misfit phenomenon softens as it can be clearly seen from the following figures. FIB/STEM analysis of the as-grown Au-Ni multilayers also proved the immiscibility of Au and Ni metals in line with main purpose of using this material-pair in order to observe a shear-induced mixing and its effect on friction.

### Characterization of Au-Ni multilayers subsequent to friction tests

In order to analyze microstructures of the worn/deformed Au-Ni multilayers after friction tests and to realize a comparison with results for the as-grown multilayers, cross-sectional imaging by FIB milling has been used. Micrographs of each Au-Ni multilayer system undergoing reciprocating sliding for 100 cycles under UHV conditions are shown in Fig. 1e–h. The HAADF STEM cross-section of the worn multilayer with a layer thickness of 10 nm is depicted in Fig. 1e. We observed that the first 8 layers are plastically deformed and possess a shear-induced or “mixed” microstructure while the remaining 92 layers are still unmixed. A reduction in the thickness of the upper layers which are adjacent to the deformed, intermixed region is visible and at the very outermost surface, we also found a zone, where individual Au and Ni structures are not distinguishable in the STEM. As we show later, a metastable AuNi (weakly ordered, nanocrystalline) alloy has been formed by shearing the multilayers which does not exist in the binary phase diagram at lower temperatures^29^. The intermixed zone has a thickness of 40–100 nm for the multilayer with 10 nm thickness (1e), and is slightly thicker for the sample with 20 nm layer thickness (1 f). The microstructure of the 50 nm multilayer sample shown in Fig. 1g differs from the 10 and 20 nm samples in so far as only 5 layers have been mixed but fragments of the individual layers can still be distinguished in the intermixed zone. Finally, the thickest multilayer structure with a layer thickness of 100 nm resulted in a thinning (by 25–30% in the very end, and 50–55% in the middle of the wear track) of the topmost Au layer; in other words, only first Au layer is plastically deformed during sliding for 100 cycles and subsequent layers are unaffected under these sliding conditions.

**Figure 2:**
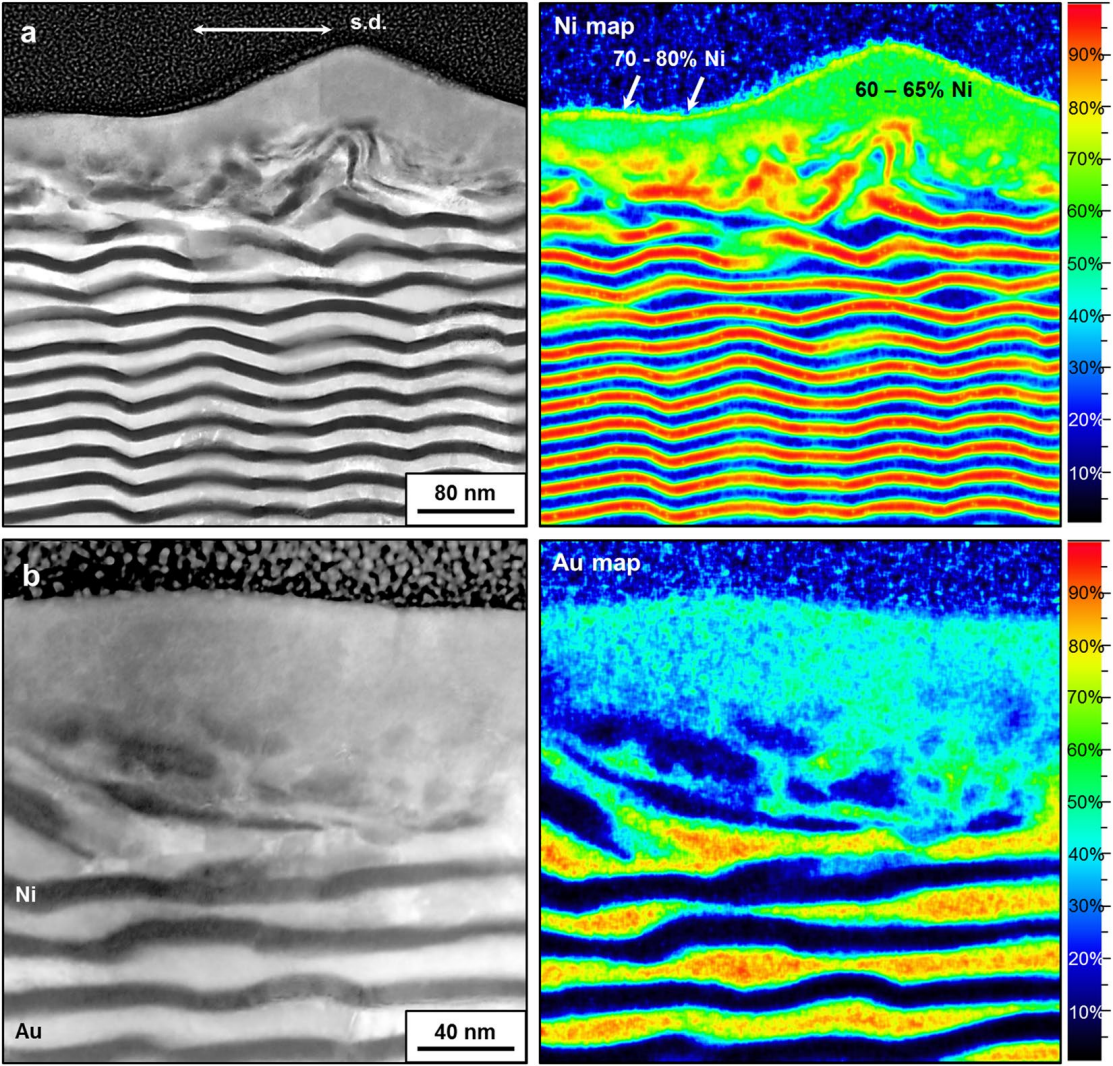
Characterization of Au-Ni multilayers with the layer thickness of 10 nm subsequent to the friction test on different regions of the intermixed tribolayer. (**a**) HAADF STEM cross-section parallel to the sliding direction (left), and corresponded EDXS map showing atomic concentrations of Ni (right); (**b**) HAADF STEM cross-section parallel to the sliding direction (left), and corresponded EDXS map showing atomic concentrations of Au (right).

In order to further analyze the worn structures and investigate the sub-surface chemistry, (S)TEM imaging, operated in the HAADF mode, in combination with energy dispersive X-ray spectroscopy (EDXS) analyses have been performed on multilayer samples. The results of the 10 nm sample are presented in Fig. 2. In these micrographs the presence of two zones becomes more obvious. On the first 40 nm from the surface, a layer is present where separated areas of Au and Ni are not found (fully mixing). Below, we found a zone that consists of mixed fragments of the former layers. Thus, the thickness of the intermixed tribolayer varies from 40 to 100 nm for these cases. The quantified EDXS maps on the right-hand side in Fig. 2 represent the atomic concentration of Ni (Fig. 2a) and Au (Fig. 2b) of the corresponding (S)TEM cross-section images on the left-hand side. By shearing, we obtained a ‘fully mixed’ structure consisting 60–65 at% of Ni in Au. Interestingly, the zone ‘within the first 10 nm from the surface’ is a more favorable place for ultrafine Ni grains to reside in most of the cases according to the EDXS maps (see white arrows in Ni map of Fig. 2a).

**Figure 3:**
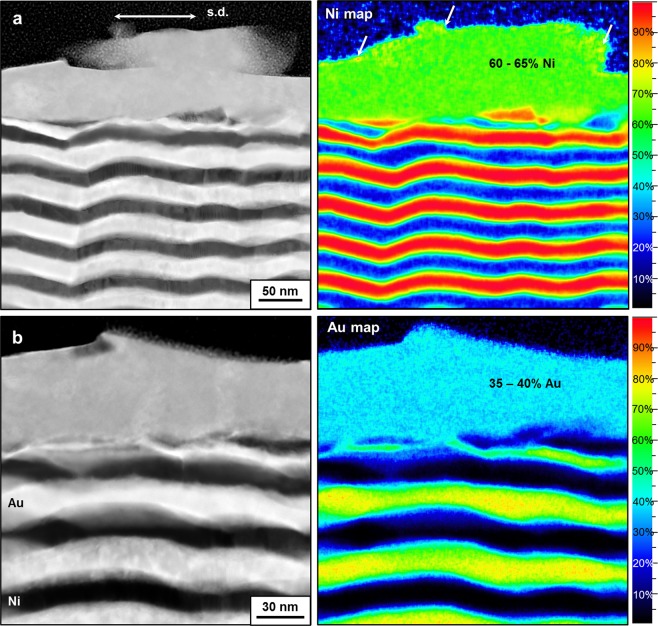
Characterization of Au-Ni multilayers with the layer thickness of 20 nm subsequent to the friction test on different regions of the intermixed tribolayer. (**a**) HAADF STEM cross-section parallel to the sliding direction (left), and corresponded EDXS map showing atomic concentrations of Ni (right); (**b**) HAADF STEM cross-section parallel to the sliding direction (left), and corresponded EDXS map showing atomic concentrations of Au (right).

The structure and chemical analysis for the multilayer system with the layer thickness of 20 nm are depicted in Fig. 3. While the observed structure already shown in Fig. 2 suggests that fully mixing is possible between Au and Ni phases through shearing, Fig. 3 also indicates this process as found by performing HAADF (S)TEM cross-section imaging with detailed EDXS mapping. Differently from the 10 nm sample, the distribution of Au and Ni in the intermixed tribolayer, seems to be more homogeneous, however, also giving a mean value of 35–40 at% Au and 60–65 at% Ni, respectively. Again, ultrafine Ni-grains tend to be present at the outermost surface (white arrows in Ni map of Fig. 3a). Compared to Fig. 2, the images presented in Fig. 3 demonstrate an increase in the thickness of the mixed layer that varies from 45 to 120 nm which also means that the thickness of the intermixed tribolayer increases slightly with increasing layer thickness of the multilayer system and this can be attributed to the initial hardness of the multilayer samples (see Hall-Petch relation in Supplementary Fig. S3).

**Figure 4:**
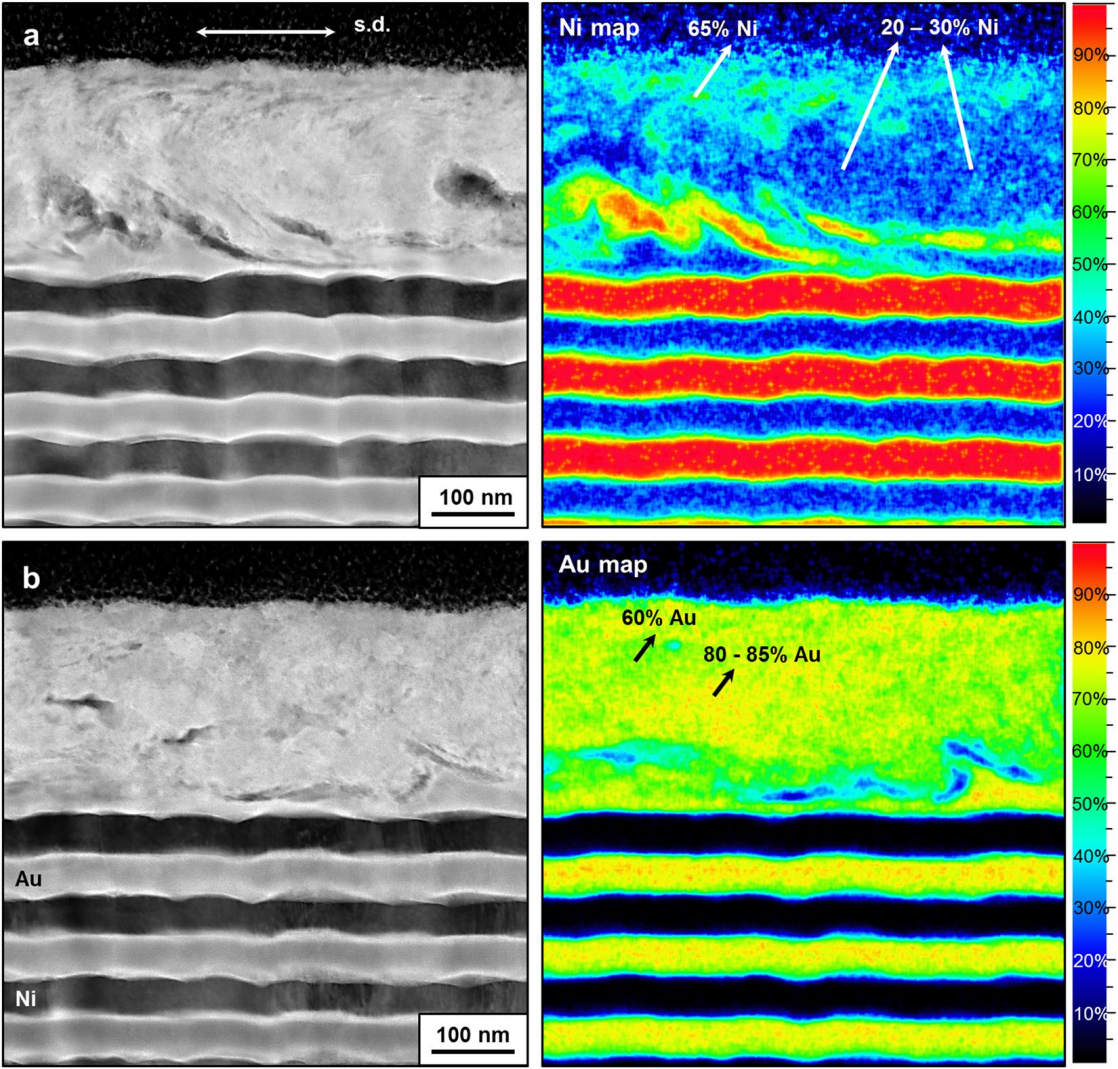
Characterization of Au-Ni multilayers with the layer thickness of 50 nm subsequent to the friction test on different regions of the intermixed tribolayer. (**a**) HAADF STEM cross-section parallel to the sliding direction (left), and corresponded EDXS map showing atomic concentrations of Ni (right); (**b**) HAADF STEM cross-section parallel to the sliding direction (left), and corresponded EDXS map showing atomic concentrations of Au (right).

The shear-induced microstructure with the layer thickness of 50 nm is presented in Fig. 4. Here layers can still be separated in the mixed region. The atomic concentration of Au in the ‘partially mixed’ tribolayer is much higher than that of Ni (mean values: 70 at% Au; 30 at% Ni). This is in contrast to the 10 nm and 20 nm samples where we observed that the layers are fully mixed (Fig. 2–3); nevertheless, this result might be reasonable due to the fact that there were 5 layers mixed from which three were Au and two were Ni layers, respectively. The thickness of the partially mixed layer is around 200 nm, supporting our former finding that the thickness of the intermixed tribolayer increases with increasing individual layer thickness of the multilayer system.

### Frictional behavior of Au-Ni multilayers

**Figure 5:**
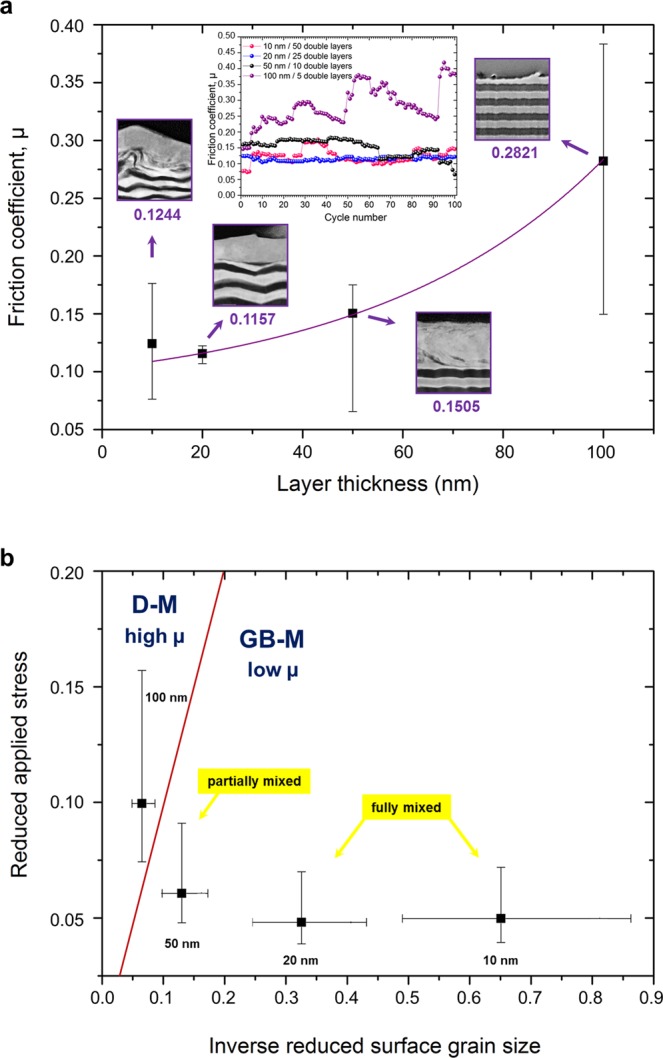
Frictional behavior of Au-Ni multilayers and relationship between microstructure, deformation mechanism and friction. (**a**) Dependence of the COF on the layer thickness. Error bars are determined by the fluctuations in friction during sliding, which are demonstrated by the inset figure. The mean values of friction coefficients are 0.1244, 0.1157, 0.1505 and 0.2821 for layer thickness of 10 nm, 20 nm, 50 nm and 100 nm respectively. **(b)** Map for dominant deformation mechanisms (grain boundary, GB- or dislocation, D-mediated) related with the friction force (µ, coefficient of friction) in terms of the reduced applied stress and the surface grain size which is adapted from ref.^25^. Data points are calculated based on the mean values of Au and Ni using correspondent equations (see the text) and the error bars depict the individual values for Au and Ni. The model predicts that multilayer systems with a layer thickness of 10 nm, 20 nm and 50 nm fall into the regime which belongs to the grain boundary-mediated deformation with low friction whereas 100 nm sample with higher friction should be connected to the dislocation-mediated deformation.

Fig. 5a shows the mean friction coefficient values for multilayer systems as a function of layer thickness. Interestingly, we found a significant increase of the coefficient of friction (COF) with increasing layer thickness which is in agreement with the previous experimental observations for Cu-Ni multilayers^30^. Please note that the values of the error bars are very different due to the different stability of friction behavior throughout sliding, as depicted in the inset of the figure.

## Discussion

The experiments clearly demonstrate that changing the layer thickness leads to very distinctive microstructural evolution for each Au-Ni multilayer system. While ‘partial mixing’ is observed for the multilayer sample with the layer thickness of 50 nm which is in line with the previous experiments^17,18^, a novel, fully mixed material is found for thinner samples which is most likely a metastable ‘AuNi’ phase. According to the binary phase diagram of the Au-Ni system^29^, a miscibility gap can only exist at higher temperatures (above a critical temperature, 810.3 °C) because of the fact that in order for two metals to form a solid solution, they must crystallize in the same lattice structure and have similar chemical properties (i.e. similar electronegativity and atomic radius). Hence, the miscibility gap at lower temperatures (below 810.3 °C) leads to the decomposition, and the AuNi solid solution separates into two FCC structural phases below the critical temperature. In other words, these metals do not create an alloy structure under normal circumstances and a separation gap is formed. Because of the immiscibility of this metal pair at lower temperatures, experimental studies are restricted based on forming a thermodynamically stable alloy structure. Recently, Swiatkowska-Warkocka *et al*. has showed that a metastable AuNi alloy with 55 at% of Ni in Au can be produced via a method ‘pulsed laser irradiation’ of colloidal nanoparticles by overheating and rapid quenching^31^. It is therefore surprising that under shear it appears to be possible to also form a metastable AuNi alloy which lowers the friction force of the system. To verify the presence of the proposed alloy structure, a high-resolution (HR) TEM study was also performed on the 10 nm sample (see supplementary Fig. S6). The HRTEM analysis confirms the formation of a new, stable nanocrystalline microstructure where individual layers or fragments of Au and Ni cannot be identified. In addition, since the calculated diffraction intensities of 0.2180–0.2197 nm^−1^ via selected area electron diffraction (SAED) analysis on the fully-mixed region might strongly correlate with the value of 0.22 nm^−1^ for the AuNi alloy structure, it can be argued that the observed lattice spacing is certainly close to the expected one for the AuNi alloy (Supplementary Fig. S6). On the other hand, sliding of samples with a larger layer thickness and also larger grain size according to the Scherrer equation^32^ (see supplementary Table S3) did not result in mechanical mixing; in the latter case only deformation of the topmost Au layer took place (the case for 100 nm sample). Those observations are eventually caused by deformation mechanisms that strongly depend on grain sizes below and above their critical value called the strongest size^33^. This is approximately the smallest grain size which allows dislocation movement to continue (i.e. intragranular sliding) and thus can be assumed as the equilibrium distance between two edge dislocations. Hence, intergranular sliding should predominate below this size. In other words, the dominant deformation mechanism should transform from bulk to interface below this critical value, since the number of grains raises and dependently, grain boundaries become more dominant in the sub-surface^33^.

Starting from this point of view, the deformation mechanisms have been carefully studied according to the initial surface grain size *d* and the dislocation splitting distance *r* for each multilayer system^25,34^. A recent model by Argibay and coworkers^25^ attempts to relate a previously published^34^ deformation mechanism map to the friction force and predicts that there should be a critical grain size which controls deformation mechanism on the sub-surface from grain boundary- to dislocation-mediated plasticity.

To test the model we determine the critical grain size using the definition for the dislocation splitting distance *r* found in Yamakov *et al*.^25,34^:

1 $$r=\frac{{r}_{0}}{1-{\sigma }_{a}/{\sigma }_{\infty }}$$

*σ*_*a*_ denotes the applied stress and ${\sigma }_{\infty }=2{\gamma }_{sf}/b$ is the critical shear stress of the material. *r*_0_ is the equilibrium dislocation splitting distance at zero stress which is defined as:

2 $${r}_{0}=\frac{(2+v)G{b}^{2}}{8\pi (1-v){\gamma }_{sf}}$$

where *γ*_*sf*_ is the stacking fault energy (SFE), *v* is the Poisson’s ratio, *G* is the shear modulus, and *b* is the Burgers vector for the dislocation. *σ*_*a*_/*σ*_*∞*_ can be utilized as the reduced stress parameter and the applied stress *σ*_*a*_ on the sliding surface is calculated using the Hamilton contact model as follows:

3 $${\sigma }_{a}=\frac{3{F}_{n}}{2\pi {a}^{2}}[\frac{1-2v}{3}+\frac{(4+v)}{8}\pi \mu ]$$

here *F*_*n*_ refers the applied load, *μ* is the friction coefficient and *a* is the contact radius calculated by the Hertzian contact model.

We calculated the values for Au-Ni multilayer systems with different layer thickness, and the map for the dominant deformation mechanisms related with the friction force, reduced applied stress and surface grain size is plotted in Fig. 5b. The model predicts that multilayer systems with a layer thickness of 10 nm, 20 nm and 50 nm fall into the regime which belongs to the grain boundary-mediated deformation with low friction. Not explained by the model is the difference in friction between the 50 nm sample and thinner multilayers that we might ascribe to the newly formed phase at the very surface showing the lowest friction (10 nm and 20 nm samples). In the previous section we have already shown modifications in microstructures after exposing materials to shear; namely, they resulted in a process including partially and fully mixing within the more stable microstructures. TEM/HRTEM analyses even reveal that ultrafine grains exist in the intermixed tribolayer which would be responsible for lower friction. It can be argued that refined grains lead to an increased number of grain boundaries which hinder the transmission of dislocations; and therefore, shearing occurs at the newly formed grain boundaries behaving as an incommensurate, low shear contact. Although latter analysis is able to explain our friction data and the observed microstructural evolution reasonably well, one should consider that the analysis is a modified version of the deformation model developed by Yamakov *et al*.^34^, where also a distinction between perfect (complete) and partial (incomplete) slip deformations of low SFE and high SFE FCC metals was made. However, only perfect dislocations have been considered in the proposed model^25^ without incorporating partial dislocations. In fact, complete and incomplete dislocations usually cooperate with each other to manage the deformation behavior such as transition from conventional to partial slip at the dislocation-splitting line. Partial dislocations can therefore be the key to inhibit the propagation of dislocations across the grains since the stacking faults transect the grains when they are sufficiently small. Hence, considering the low and high stacking fault energies of Au (𝛾_𝑠𝑓_ = 45 mJ/m^2^) and Ni (𝛾_𝑠𝑓_ = 128 mJ/m^2^), we can refer to the possible transition from the partial dislocation- to grain boundary-mediated deformation during sliding of the 50 nm sample arising from the formation of the vortex-like structures (where the grain size is assumed to be on the fringe of critical size) as well as its inevitable impact on friction. Note that grain boundary-mediated scenarios included in Yamakov’s deformation model^34^ have also been suggested for the plasticity of other material systems such as the deformation mechanism of the Al-Cu joint under tensile loading^35^ and grain size dependence of tensile behavior in nanocrystalline Ni-Fe^36^. Also, atomistic simulations are able to shed further light on grain boundary-induced deformation (see eg^37,38^). On the other hand, the multilayer system with a layer thickness of 100 nm resulted in different behavior which means that dislocation-mediated deformation should be in charge for the observed higher friction behavior. The experimental observation shown in Fig. 1h is also consistent with this argumentation due to the existence of a material pile-up at the end of the wear track subsequent to shearing process. Rather than a mixing, thinning of the uppermost Au layer has been observed in this sample which demonstrates a bulk-accumulation of dislocations as long as sliding occurs in mentioned conditions. According to the model above, dislocation-mediated plasticity would be expected if the applied shear stress becomes greater than the critical value (${\sigma }_{a}/{\sigma }_{\infty }\ge 1$); however, in this presented case, the reduced stress parameter has been calculated as 0.0995 which is interestingly lower than 0.5. We can infer that the model applied to Au-Au contacts^25^ is in accordance with our results for thinner samples since more stable microstructures within the refined grains have been reached at the end of the process. Nonetheless, for the non-mixing case, even though the result shows a dislocation-mediated deformation and high friction in the map (Fig. 5b), the value for the reduced stress parameter is less than the threshold value. The discrepancy might stem from the fact that our material system consists of Au and Ni and the counter body is ruby instead of a pure metallic contact. Moreover, the large difference in the stacking fault energies of Au (𝛾_𝑠𝑓_ = 45 mJ/m^2^) and Ni (𝛾_𝑠𝑓_ = 128 mJ/m^2^) does not allow us to determine an exact value of the 𝛾_𝑠𝑓_ for the shear-mixed Au-Ni alloy in thinner samples, as well as for the Au-Ni composite system in the case of 100 nm sample. Thus, we averaged these values at the main data point of the graph in Fig. 5b whereas we used the individual values of Au and Ni as the error bars (see supplementary Table S1), resulting in the error bars shown (see Fig. 5b).

In conclusion, by performing sliding experiments with carefully prepared Au-Ni multilayer samples under well-defined UHV conditions, we showed that the individual layer thickness of Au-Ni multilayer systems has a strong impact on the resulting friction force due to a transition of the dominant deformation mechanism close to the surface. We might also observe for the first time the formation of a shear-induced metastable AuNi alloy within the more stable microstructure in the fully mixed tribolayer of the 10 nm and 20 nm samples, corresponding to the lowest observed friction forces. A possible route to the reformation of the new phase is the initial formation of ultrafine grains to increase the number of grain boundaries during sliding and consequently, grain boundary-mediated deformation resulted in the low friction coefficient. When increasing the layer thickness of the multilayer system to above 50 nm, a partially mixed, Au rich layer is formed instead. When further increasing the layer thickness, rather than a partially or fully mechanical mixing, the thinning of the uppermost Au layer eventuated by plowing and accumulating dislocations at the end of the wear track. Dislocation-mediated deformation is considered to be dominant in this multilayer sample and higher friction has been obtained while only first Au layer is plastically deformed.

## Methods

### Sample preparation

*Ex-situ* cleaning of Si wafers (100) took place in two steps. A washing-up liquid (a tenside solution) was used as the pre-cleaner in an effort to achieve rough cleaning of the substrates whereas an acetone solution was applied in an ultrasonic bath for 2 hours for the final stage. Plasma etching was applied inside the physical vapor deposition (PVD) chamber (Leybold Z550) for 2 minutes to clean and activate the substrate surface at an atmosphere of 0.5 Pa Ar in 6 N and a power of 500 W. Subsequently, the growth of Ni and Au layers with well-defined microstructures on Si substrates took place via magnetron sputtering at 0.4 Pa Ar in 6 N purity atmosphere by using 75 mm diameter elemental targets (see supplementary Table S2). The individual layer thickness of the samples was 10 nm, 20 nm, 50 nm, 100 nm, respectively and the number of layers was changed from 100 to 10 thus fixing total layer thickness at 1 µm. In order to prevent the structure from possible oxidation, the topmost layer was chosen Au whereas the first layer on the substrate was Ni for all studied samples.

### XRD analysis

The measurements were carried out using CuK_α1/2_ radiation in Bragg-Brentano geometry at a Seifert PAD II diffractometer equipped with a Meteor 1D detector. In all measurements, diffraction peaks (111), (200) and (222) of Au were present as principal peaks and there seem no reflections from the Ni layers since Ni (111) and Au (200) reflections have almost the same diffraction angle. The broadening of the peaks was noticeable as the number of double layers increases (see Supplementary Fig. S1), which also indicates the lower grain size with decreasing layer thickness (see Supplementary Table S3).

### AFM analysis

A commercial AFM (Veeco Dimension V, now Bruker) was utilized in contact mode to perform roughness analysis on the uppermost layers of Au-Ni multilayer samples as shown in Supplementary Fig. S2. Si cantilevers (Budget sensors uncoated Tap300 series with the radius of curvature less than 10 nm, a resonant frequency of 300 kHz and a spring constant of 40 N/m, which are appropriate for intermittent contact mode) were used during the measurements.

### Nanoindentation analysis

A ‘Hysitron Ti 950 Triboindenter’ (Bruker) nanoindenter was utilized to determine the hardness of the as-grown Au-Ni multilayer samples by constituting the indentation load-displacement curves. A Berkovich type diamond triangular pyramid indenter was used in the measurements and different indentation loads from 2 to 8 mN were applied to the multilayer samples. The results are presented in Supplementary Fig. S3.

### Microfriction experiments in UHV

Surface cleaning of the multilayer samples was achieved prior to each friction test via Ar ion sputtering in the XPS chamber (PHI5000 Versaprobe II), which is connected to the microtribometer and allows directly transferring the sample between the chambers. For cleaning, the Ar ion beam was rastered over an area of 5 mm × 5 mm on the surface of the sample for approximately 0.5 min (which would be equivalent an amount of less than 1 nm of removal) using an accelerating voltage of 2 kV in the XPS chamber. Supplementary Fig. S4a represents the corresponding surface overview before sputtering in which carbon (C) and oxygen (O_2_) peaks are visible whereas the clean surface achieved by the ion bombardment (removal of C and O_2_) is shown in Supplementary Fig. S4b. Subsequently, friction tests on Au-Ni multilayer structures were run via the UHV microtribometer at a base pressure of 10^−7^ Pa^39^. An inert ruby sphere with the diameter of 3 mm was slid on the surfaces of Au-Ni multilayers at a constant normal load of 1 mN and a constant sliding velocity of 33 µm/s, and experimental setup of interest is presented in Supplementary Fig. S5. The cantilever was fixed while the sample was being scanned with the help of a piezo actuator, and friction force signals were recorded during originating wear track (plastic deformation) on a sliding distance of 100 µm within a reciprocating motion for 100 cycles.

### FIB/SEM analysis

For cross-sectional microstructure evolution, multilayer samples were prepared parallel to the sliding direction via FIB milling (FEI Helios Nanolab 650, now ThermoFisher, operated at 30 kV) following common procedures for the *in-situ* lift out technique^28^. And subsequently, the cut lamellas were imaged in the HAADF mode via the STEM detector in the same instrument.

### TEM/HRTEM and STEM-EDXS analyses

In order to further analyze the worn microstructures of Au-Ni multilayer samples with atomic resolution, TEM/HRTEM analyses were applied via a Philips CM200 FEG/ST operated at an acceleration voltage of 200 kV. A SAED aperture was also utilized to examine the crystal orientations in the sub-surface. The images of interest were presented in Supplementary Fig. S6. TEM in STEM mode in combination with the EDXS mapping were also performed to investigate the sub-surface chemistry of Au-Ni multilayer samples after sliding by using a FEI Osiris ChemiSTEM, with an acceleration voltage of 200 kV. Images were obtained in bright field (BF), dark field (DF) and HAADF modes by providing different contrasts.

## Supplementary information

Supplementary Information for the manuscript

## Data Availability

The datasets generated and analyzed during the current study are available from the corresponding author on request and will be made available via the KIT OpenData repository.
