# Supplementary material for: Low friction of metallic multilayers by formation of a shear-induced alloy
Source: Sci Rep. 2019 Jul 1;9:9480. doi: 10.1038/s41598-019-45734-7 (PMC6602972; doi:10.1038/s41598-019-45734-7)
Supplement: Supplementary file 1 — Supplementary Information for the manuscript [file 41598_2019_45734_MOESM1_ESM.pdf]

Supplementary Information for

Low friction of metallic multilayers by formation of a shear-induced alloy

Ebru Cihan, Heike Störmer, Harald Leiste, Michael Stüber, Martin Dienwiebel*

* e-mail: martin.dienwiebel@kit.edu

Supplementary Table S1 | Experimental data and calculated parameters for studied model

Au-Ni (ν = 0.375; E = 125 GPa; G = 51.5 GPa; b = 2.69 A; γ_{sf} = 86.5 mJ/m^2)

d (nm)	µ	r_0 (nm)	r_e (nm)	r_0 / d	a (µm)	σ_∞ (MPa)	σ_a (MPa)	σ_a / σ_∞
10	0.124352	6.51	6.851	0.651	2.4	643	32	0.0498
20	0.115686	6.51	6.84	0.3255	2.47	643	31	0.0482
50	0.1505	6.51	6.93	0.1302	2.58	643	39	0.0607
100	0.28212	6.51	7.23	0.0651	2.52	643	64	0.0995

Au (ν = 0.44; E = 79 GPa; G = 27 GPa; b = 2.88 A; γ_{sf} = 45 mJ/m^2)

d (nm)	µ	r_0 (nm)	r_e (nm)	r_0 / d	a (µm)	σ_∞ (MPa)	σ_a (MPa)	σ_a / σ_∞
10	0.124352	8.63	9.3	0.863	2.66	312	22.53	0.072
20	0.115686	8.63	9.28	0.4315	2.72	312	21.84	0.07
50	0.1505	8.63	9.494	0.1726	2.85	312	28.44	0.091
100	0.28212	8.63	10.24	0.0863	2.79	312	48.94	0.157

Ni (ν = 0.31; E = 170 GPa; G = 76 GPa; b = 2.49 A; γ_{sf} = 128 mJ/m^2)

d (nm)	µ	r_0 (nm)	r_e (nm)	r_0 / d	a (µm)	σ_∞ (MPa)	σ_a (MPa)	σ_a / σ_∞
10	0.124352	4.9	5.1	0.49	2.27	1028	40.65	0.0395
20	0.115686	4.9	5.098	0.245	2.32	1028	40	0.0389
50	0.1505	4.9	5.147	0.098	2.43	1028	49.39	0.048
100	0.28212	4.9	5.293	0.049	2.38	1028	76.43	0.0743

Supplementary Table S2 | Sputtering conditions for Ni and Au layers

Thickness (nm)	Sputter time (s) 300 W HF, Ni	Sputter time (s) 20 W DC, Au
100	142	163
50	71	82
20	28	33
10	14	16

Supplementary Table S3 | Grain size approximation via the XRD data using the Scherrer equation:

$\Delta(2\theta) = \frac{K\lambda}{Lcos\theta}$)

Au(111)

Spacing (nm)	Measured broadening at FWHM	Instrumentation broadening	Line broadening $\Delta(2\theta)$ (MB - IB)	Scherrer constant, K	X-ray wavelength, λ (Å)	Bragg angle, 2θ	Grain size (L) (nm)
10	0.8204°	0.07°	0.7504°	0.89	1.54056	38.3°	11.08
20	0.575°	0.07°	0.505°	0.89	1.54056	38.3°	16.47
50	0.3571°	0.07°	0.2871°	0.89	1.54056	38.3°	28.97
100	0.2406°	0.07°	0.1706°	0.89	1.54056	38.3°	48.75

Ni(111)

Spacing (nm)	Measured broadening at FWHM	Instrumentation broadening	Line broadening $\Delta(2\theta)$ (MB - IB)	Scherrer constant, K	X-ray wavelength, λ (Å)	Bragg angle, 2θ	Grain size (L) (nm)
10	0.8273°	0.07°	0.7573°	0.89	1.54056	44.5°	11.22
20	0.4855°	0.07°	0.4155°	0.89	1.54056	44.5°	20.43
50	0.2771°	0.07°	0.2071°	0.89	1.54056	44.5°	40.99
100	0.2280°	0.07°	0.158°	0.89	1.54056	44.5°	53.71

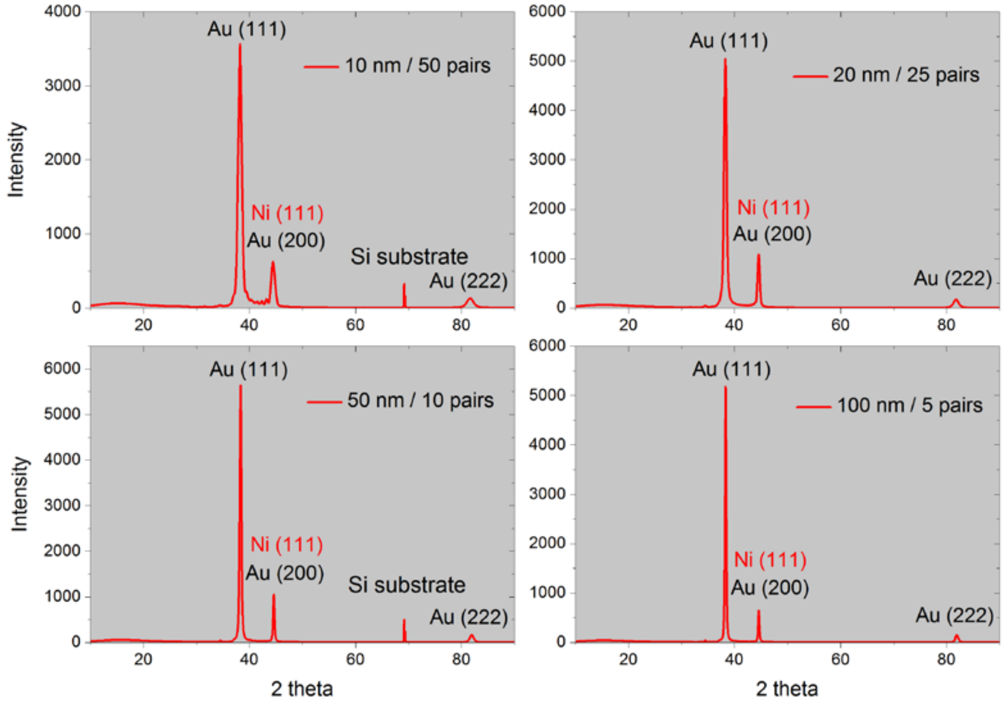

Supplementary Figure S1 | XRD profiles of individual Au-Ni multilayer samples. The diffraction peaks (111), (200) and (222) of Au are present as principal peaks and there seem no reflections from the Ni layers since Ni (111) and Au (200) reflections have almost the same diffraction angle. The broadening of the peaks is noticeable as the number of double layers increases, which also indicates the lower grain size with decreasing layer thickness according to the approximation with Scherrer equation [1].

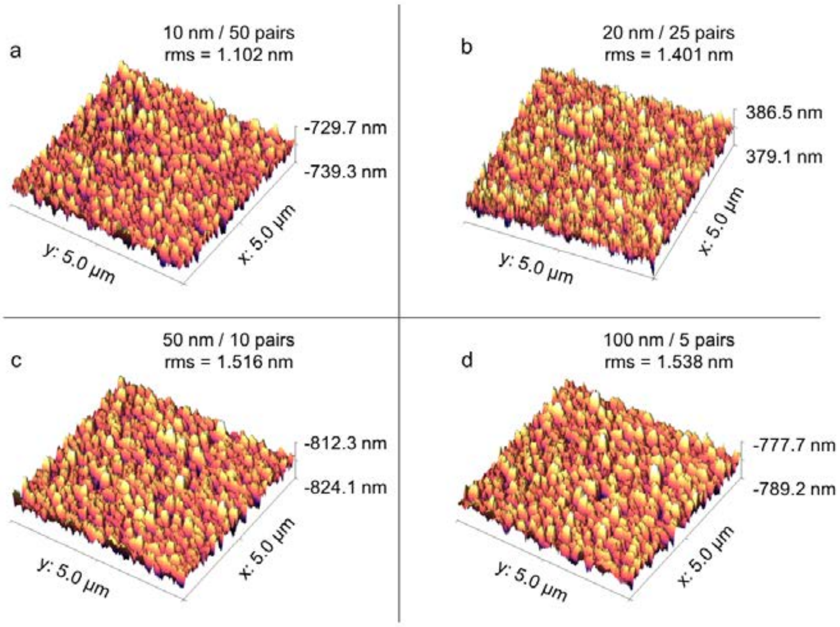

Supplementary Figure S2 | AFM topography analysis of the topmost layers of as-deposited Au-Ni multilayers, as a function of individual layer thickness. Roughness values are **(a)** 1.102 nm for 10 nm-multilayer sample; **(b)** 1.401 nm for 20 nm-multilayer sample; **(c)** 1.516 nm for 50 nm-multilayer sample; **(d)** 1.538 nm for 100 nm-multilayer sample. The roughness values of topmost layers slightly increases with increasing layer thickness.

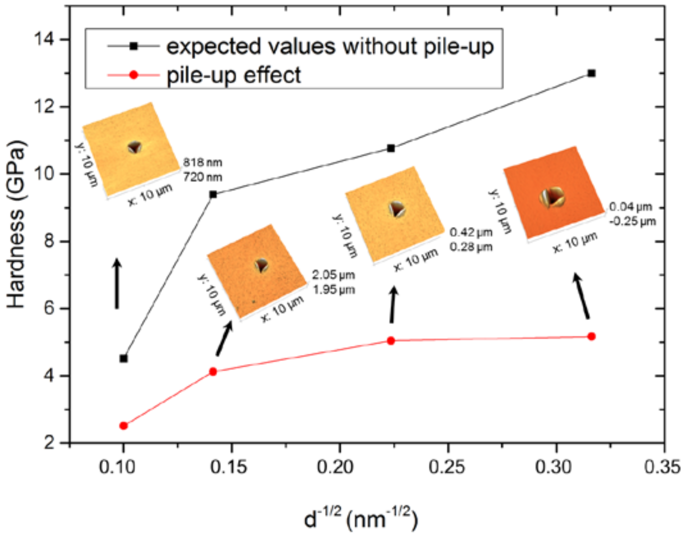

Supplementary Figure S3 | Nanoindentation measurements on Au-Ni multilayer samples. Indentation hardness as a function of the interlayer spacing of the multilayers at 8 nN applied load. An increase in the layer thickness leads to decrease in hardness of the multilayers by 58.3 ± 7 %, from 10 to 100 nm sample (without pile-up), which is in agreement with the Hall-Petch relationship. Note however that significant amount of pile-up is observed in our measurements. Hence, in order to eliminate the pile-up effect and correct the hardness of multilayer samples, a simple geometrical approach is used based on the determination of the area of the triangle indent [2].

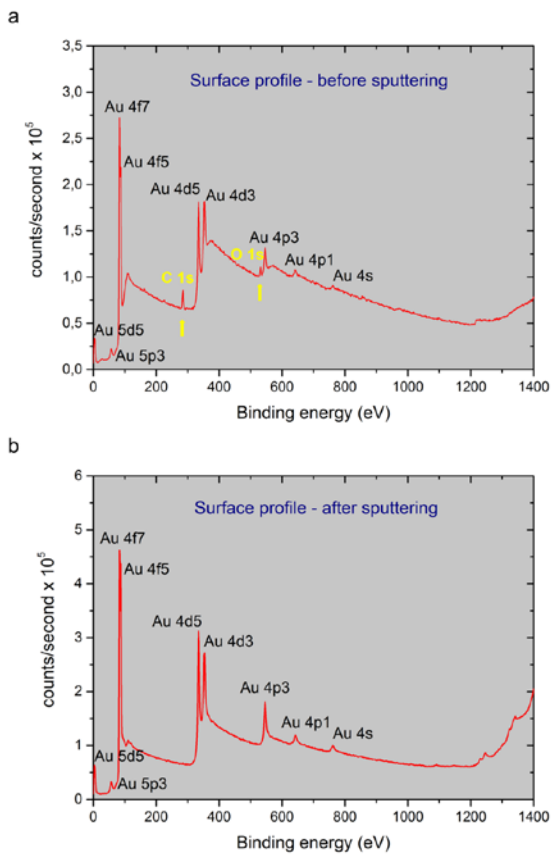

Supplementary Figure S4 | XPS surface profiles of Au-Ni multilayer samples. (a) C and O_2 peaks are present on the topmost surface when Au-Ni multilayer sample is introduced into the XPS chamber from air; (b) C and O_2 are removed from the topmost surface by Ar ion milling (sputtering), which means that surface cleaning can be achieved prior to the friction test in the UHV microtribometer.

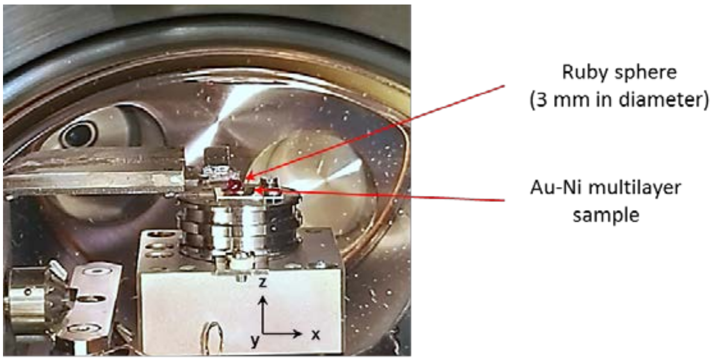

Supplementary Figure S5 | Picture of the experimental setup used in the friction tests. Note that the reciprocating sliding is always applied in the y direction while the sample is positioned in the x direction and mechanical stress is loaded in the z direction.

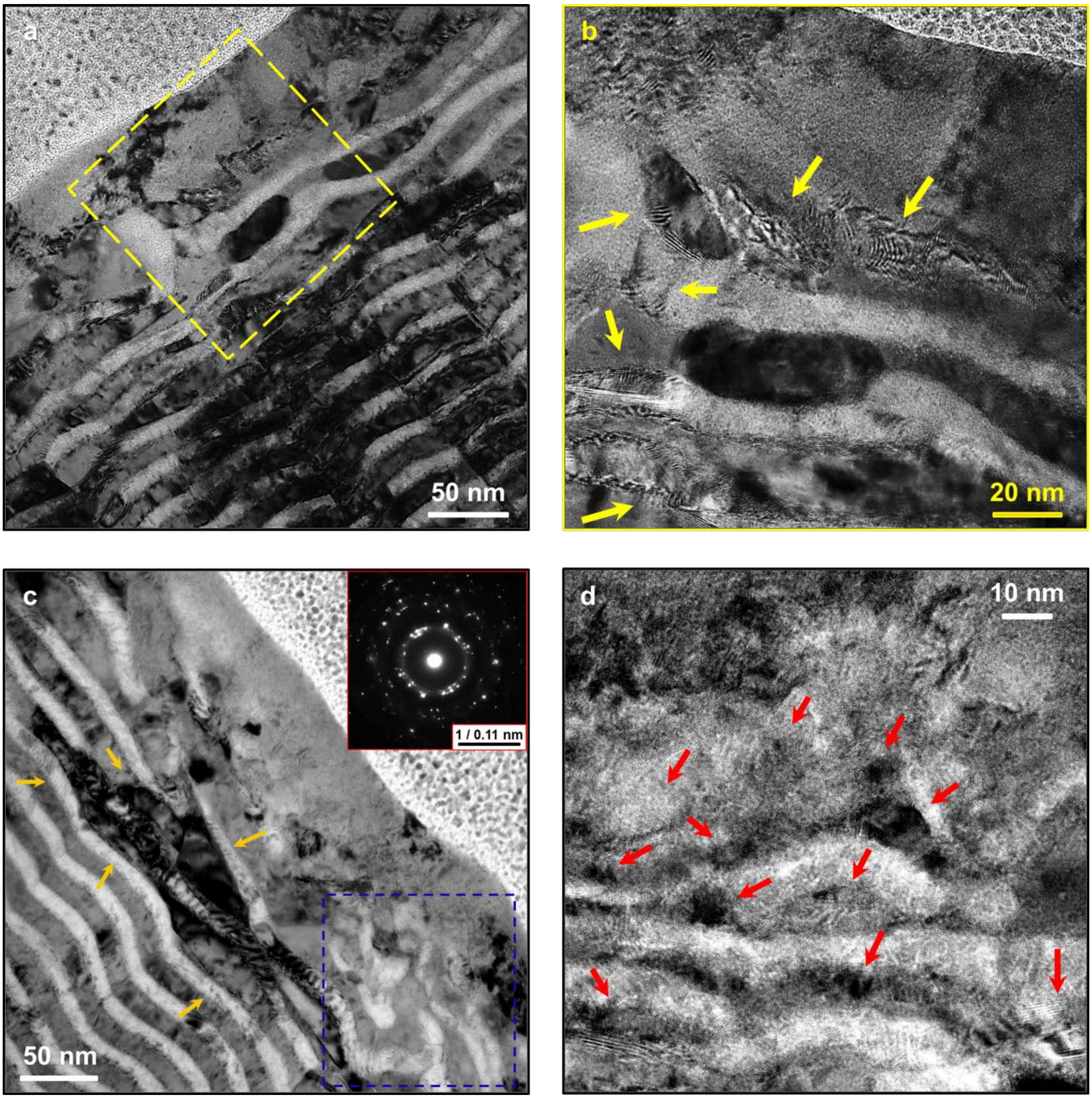

Supplementary Figure S6 | TEM images of worn 10 nm multilayer sample. The images show intermixed nanocrystalline zones with ultrafine Au, Ni and/or AuNi grains, and coarsened grains between not completely deformed layers beneath this zone. Crystal defects are visible both in the intermixed zone and between the individual Au and Ni layers. **(a)** An overview to the corresponding surface in which coarsened (probably Au) grains situated between not completely deformed (probably Ni) layers right below the mixed nanocrystalline region can be distinguished. **(b)** Close-up image which is highlighted with the yellow dashed square in **(a)**. Yellow arrows indicate coarsened grains in different contrasts. Since the main contrast mechanism is controlled by the Bragg-diffraction, individual grains change their contrast (enhance contrast and become visible, or lower contrast and become invisible) depending on the tilt angle. Defects can become more definable in this resolution. **(c)** Again, the image shows coarsened grains beneath the intermixed nanocrystalline zone remarked with yellow arrows. In blue dashed square, deformed but not mixed-individual (probably Ni) fragments can be seen. Beyond, fully-mixed, nanocrystalline (grayish) zone can easily be distinguished on top of the surface and a SAED pattern taken from this specific zone is shown with the inset of the figure. **(d)** HRTEM image taken in between the mixed and non-mixed regions; non-mixed regions have however shear-induced deformed layers. The huge amount of the residual strain is the main parameter which inhibits taking a better quality image in this sample as clearly followed by the picture noise. Red arrows depict the nanocrystalline grains and more strained nanocrystals in the worn region.

Supplementary Note S1 | Grain coarsening following the process of sliding

Grain coarsening in nanocrystalline materials is driven by the excess surface free energy. In other words, grains in a material always try to minimize their surface free energies by reducing the total grain boundary energy [3]. At this point, it is worth further addressing the effect of alloying on the microstructure evolution. Alloying contributes to the more durable microstructures by refining grains as well as creating Zener pinning sites in polycrystalline surfaces. Those fine pinning sites reduce the grain boundary mobility by applying a pinning force that can eliminate the impact of driving force to trigger the mobility. In this sense, grain coarsening on the sub-surface might be prevented during mechanical alloying (fully mixing) by introducing this kind of pinning sites, also leading to the lower friction behavior as already presented in the main text. As previously mentioned, and presented in Supplementary Fig. S6a-d, TEM analysis on the 10 nm sample confirmed the formation of a more stable microstructure by grain refinement due to the mixing of Au and Ni layers near to the surface.

On the other hand, grain coarsening beneath the intermixed region has been also observed. Although residual strain has already been present in the as-grown multilayers induced by the fact of lattice mismatch between Au and Ni metals (15%), as well as the orientation dependent growth rate, TEM analysis revealed that there should still exist a huge amount of strain in the material after achieving a more stable microstructure by grain refinement (this evidence can refer to the SAED analysis mentioned in the next Supplementary Note). TEM images in Supplementary Fig. S6a-d depict coarsened grains where the process takes place between not completely deformed layers close to the intermixed region, due to that a material tends to recrystallize itself in order to reduce strain. It can be also argued that deforming the hard Ni layers by shearing, it would be possible to fold (softer) Au layers in order to agglomerate them in a grain format.

Bending the crystallographic planes leads to observing different contrasts in a TEM image due to the fact that changes in tilt angle are highly influential on the Bragg diffraction conditions. And mostly, existent crystal defects in the sample result in bending the planes. For this reason, TEM contrast study would be pretty indicative to understand the characteristics of the defect, depending on bending [4]. Although this aspect is more beyond our scientific purpose to achieve in this article, we presented TEM images of worn 10 nm multilayer sample, including different contrasts for the grains (Supplementary Fig. S6b-c). Depending on the grain orientation with respect to the incident electron beam, different grains show higher contrast (appear darker) or lower contrast (appear brighter) upon tilting the sample.

Supplementary Note S2 | TEM and diffraction analysis on the intermixed tribo-layer

To distinguish Au, Ni and AuNi alloy grains from each other, SAED analysis was carried out on the fully-mixed region; however, diffraction analysis is extremely challenging since Au and Ni have the same crystal symmetry and space groups (Fm3m) in addition to the similar lattice constants[5] (a_{Au} = 0.408 nm, a_{Ni} = 0.352 nm and a_{AuNi} = 0.38 nm) and they were splitting off in their (111) Bragg diffraction conditions ($d_{hkl,Au}$ = 0.235 nm^{-1}, $d_{hkl,Ni}$ = 0.203 nm^{-1} and $d_{hkl,AuNi}$ = 0.22 nm^{-1}) due to the high amount of strain inside the material during the measurements as shown with the inset in Supplementary Fig. S6c. Nevertheless, the calculated diffraction intensities in our measurements were varying in between 0.2180 and 0.2197 nm^{-1} which might be strongly correlated to the value of 0.22 nm^{-1} for the AuNi alloy structure.

Supplementary Note S3 | Twin boundaries and grain rotation during shearing

Since a large amount of mixing instabilities have been generated during shearing of multilayers, which has actually resulted in residual stress in the material, we were unable to properly observe the twin boundaries and grain rotation in the corresponding tribolayer. However, one would definitely expect observing twin boundaries and grain rotation during shear deformation of such a material. To prove this argument, we would like to refer to a PhD thesis's chapter by Adrien Gola (Karlsruhe Institute of Technology, 2019) [6] on the tribological loading of Cu-Au multilayers. According to his MD simulations, in addition to that vortex formation is possible between Cu and Au during shear-mixing, the boundaries of the twinning event and the rotation of grains has been observed as the shear strain increases, and this led to the creation of distinct orientations in the material system. In order to investigate the possible size effect on the rotation mechanism, he increased the size of the cell used in

simulations and as a result observed similar misoriented zones in the system. As we already indicated in the original manuscript that the vortex-like structures produced in the 100 nm Au-Cu sample during shearing [7] are actually similar to what we observed in the 50 nm Au-Ni sample, we can consider the abovementioned calculations for the shear deformation of our Au-Ni system.

Supplementary References

1. Scherrer, P., Nachrichten von der Gesellschaft der Wissenschaften zu Göttingen, *Mathematisch-Physikalische Klasse* **2** 98-100 (1918).
2. Zhou, X., Jiang, Z., Zang, H., Yu, R., Investigation on methods for dealing with pile-up errors in evaluating the mechanical properties of thin metal films at sub-micron scale on hard substrates by nanoindentation technique, *Materials Science and Engineering A* **488**, 318-332 (2008).
3. Porter, D. A., Easterling, K. E., Phase Transformations in Metals and Alloys, *Springer-Science+Business Media, B.Y.* - Second Edition, Ch. 3 (1992).
4. Williams D. B., Carter C. B., Transmission Electron Microscopy: A Textbook for Materials Science, *Springer US* - Second Edition, Ch. 24-26 (2009).
5. Foiles, S. M., Baskes, M. I., Daw, M. S., Embedded-atom-method functions for the fcc metals Cu, Ag, Au, Ni, Pd, Pt, and their alloys, *PRB* **33,** 7983-7991 (1986).
6. Gola, A., Deformation of metallic multilayers: an atomistic study of the relationship between structure and deformation mechanisms, Karlsruhe (2019).
7. Luo, Z. P., Zhang, G. P., Schwaiger, R., Microstructural vortex formation during cyclic sliding of Cu/Au multilayers, *Scripta Materilia* **107**, 67-70 (2015).
